# Case report: Persistent hypogammaglobulinemia and mixed chimerism after HLA class-II disparate-hematopoietic stem cell transplant

**DOI:** 10.3389/fimmu.2024.1397567

**Published:** 2024-07-09

**Authors:** Melanie de Gier, Ingrid Pico-Knijnenburg, Monique M. van Ostaijen-ten Dam, Dagmar Berghuis, Frans J. Smiers, Adriaan A. van Beek, Hetty Jolink, Patty M. Jansen, Arjan C. Lankester, Mirjam van der Burg

**Affiliations:** ^1^ Department of Pediatrics, Laboratory for Pediatric Immunology, Willem-Alexander Children’s Hospital, Leiden University Medical Center (LUMC), Leiden, Netherlands; ^2^ Department of Pediatrics, Division of Pediatric Immunology, Hematology and Stem Cell Transplantation, Willem-Alexander Children’s Hospital, Leiden University Medical Center (LUMC), Leiden, Netherlands; ^3^ HLA Laboratory, Department of Immunology, Leiden University Medical Center (LUMC), Leiden, Netherlands; ^4^ Department of Infectious Diseases, Leiden University Center of Infectious Diseases (LU-CID), Leiden University Medical Center (LUMC), Leiden, Netherlands; ^5^ Department of Pathology, Leiden University Medical Center (LUMC), Leiden, Netherlands

**Keywords:** allogeneic hematopoietic stem-cell transplant (HSCT), hypogammaglobulinemia, HLA class-II, mixed chimerism, B-cell phenotyping, B-cell receptor repertoire

## Abstract

Allogeneic hematopoietic stem cell transplantation (HSCT) is a curative treatment for various hematological, immunological and metabolic diseases, replacing the patient’s hematopoietic system with donor-derived healthy hematopoietic stem cells. HSCT can be complicated by early and late events related to impaired immunological recovery such as prolonged hypogammaglobulinemia post-HSCT. We present a 16-year-old female patient with sickle-cell disease who underwent HSCT with stem cells from a human leukocyte antigen (HLA) class-II mismatched family donor. While cellular recovery was good post-HSCT, the patient developed mixed chimerism and suffered from cervical lymphadenopathy, recurrent airway infections and cutaneous SLE. She presented with hypogammaglobulinemia and was started on immunoglobulin substitution therapy and antibiotic prophylaxis. B-cell phenotyping showed that she had increased transitional and naïve mature B cells, reduced memory B cells, and diminished marginal zone/natural effector cells. In-depth immunophenotyping and B-cell receptor repertoire sequencing ruled out an intrinsic B-cell defect by expression of activation-induced cytidine deaminase (AID), presence of somatic hypermutations and differentiation into IgG- and IgA-producing plasma cells *in vitro.* Immunohistochemistry and flow cytometry of lymph node tissue showed a clear block in terminal B-cell differentiation. Chimerism analysis of sorted lymph node populations showed that exclusively patient-derived B cells populated germinal centers, while only a minor fraction of follicular helper T cells was patient-derived. Given this discrepancy, we deduced that the HLA class-II disparity between patient and donor likely hinders terminal B-cell differentiation in the lymph node. This case highlights that studying disturbed cognate T-B interactions in the secondary lymphoid organs can provide unique insights when deciphering prolonged hypogammaglobulinemia post-HSCT.

## Introduction

Hematopoietic stem cell transplantation (HSCT) is a potentially curative treatment for a wide range of inherited and acquired immune and hematological diseases. Next to the aim to replace the affected hematopoietic system, a well-reconstituting immune system is pivotal for adequate long-term graft function and clinical outcome. Various factors, including match grade; graft source; conditioning regime; the occurrence of graft-versus-host-disease (GvHD); infections and chimerism shape the characteristics of immune reconstitution. While T-cell reconstitution has been studied extensively, studies on B-cell reconstitution are limited (1–3). Using a linear mixed model, our group has demonstrated that B-cell reconstitution is dependent on time post-transplant, donor age and recipient age – a host-factor –, but not on stem-cell source, match grade and conditioning regimen (4). Long-term immunological complications include selective hypogammaglobulinemia, autoimmunity and inadequate vaccination responses; all of which are associated with impaired functional B-cell recovery (5, 6).

The underlying mechanisms of hypogammaglobulinemia post-HSCT are not well understood. One important facet is the influence of cognate interactions between T and antigen-presenting cells affecting locally produced cytokines and chemokines that would normally drive terminal B-cell differentiation. Here, we describe a patient who suffered from hypogammaglobulinemia and mixed chimerism persisting for up to 7 years post-HSCT for sickle-cell disease (SCD) whom we have studied through extensive phenotyping of B-cell development in peripheral blood and secondary lymphoid organs to determine the nature of her hypogammaglobulinemia.

## Case presentation

We report a 16-year-old female patient with SCD experiencing frequent vaso-occlusive bone crises who underwent HSCT from her mismatched sibling donor. Patient and donor shared the paternal, and part of the maternal human leucocyte antigen (HLA)-haplotype. A crossover event during meiosis-I caused the patient to receive the translocated HLA-DRB1 and HLA-DQB1 alleles from the other maternal haplotype.

The patient was preconditioned with fludarabine (cumulative dose 150mg/m^2^) and dexamethasone (cumulative dose 125mg/m^2^) and developed cervical lymphadenopathy later deemed fludarabine-associated toxicity after exclusion of an infectious cause. Subsequent conditioning included fludarabine, treosulfan and anti-thymocyte globulin (cumulative dose 150mg/m^2^; 42 gr/m^2^; and 8mg/kg, respectively).Cyclophosphamide was given at day 3-4 (cumulative dose 100mg/kg), followed by mycophenolate mofetil (30 mg/kg/day) with cyclosporin A (CsA; plasma levels 150-200 μg/L) from day +5 onwards as graft-versus-host-disease (GvHD) prophylaxis. Post-HSCT her lymphadenopathy recurred and considered as toxic or reactive to fludarabine after ultrasound scan. On day 19, CsA was switched to sirolimus due to inadequate plasma levels.

**Figure 1:**
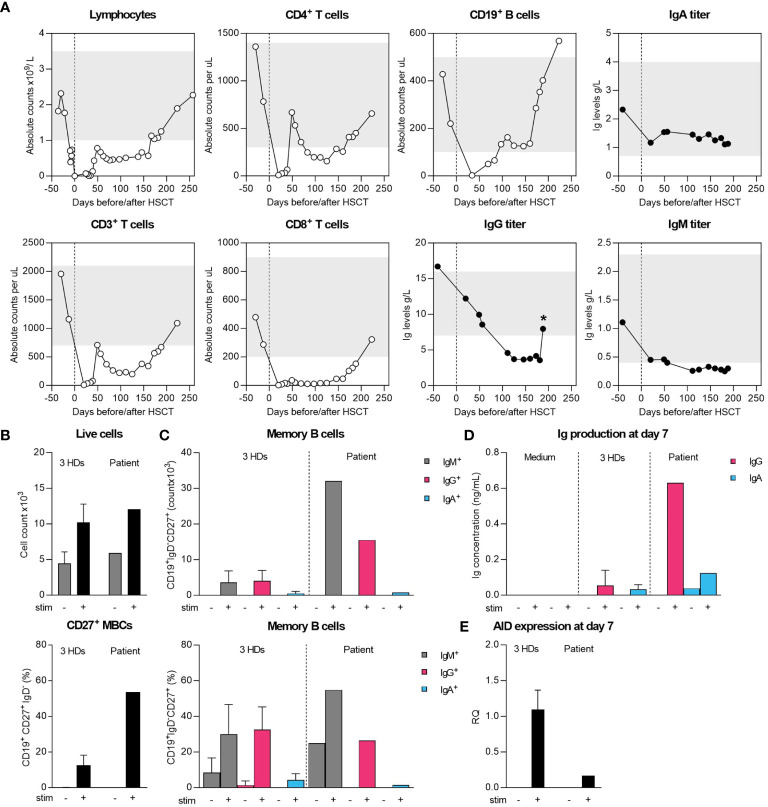
Cellular and humoral immune recovery post-HSCT and intrinsic differentiation capacity of sorted naïve B cells. **(A)** Recovery of T and B cells and serum Ig levels until patient-initiated loss-to-follow-up compared to age-matched normal range (grey bars). Star (*) indicates measurement after starting IGRT therapy. **(B, C)** Sorted naive B cells were cultured for 7 days with MEGACD40L, IL-21, IL-10 and aIgM to initiate differentiation into IgG/IgA-expressing B cells. On day 7, flow cytometry was performed to determine the absolute and relative IgM^+^, IgG^+^ and IgA^+^ B cells within CD19^+^IgD^-^CD27^+^ cells. **(D)** Supernatant collected on 7 day was used to assess IgG and IgA-levels by ELISA. **(E)** The expression of *AID* was assessed by qPCR on collected RNA from unstimulated and stimulated conditions.

The patient’s cellular recovery was normal initially with normal T- and B-cell counts (**Figure 1A**) and without GvHD nor PCR-detectable viral reactivations. However, her humoral immunity did not appear to reconstitute, with IgG levels consistently dropping until below reference values at 3 months ensuing immunoglobulin replacement therapy (IGRT) which was started at 6 months post-HSCT. Following a period of patient-initiated reduced follow-up frequency, IGRT therapy was restarted with cotrimoxazole for frequent respiratory tract infections. It appeared her cervical lymphadenopathy never fully resolved, and she developed IgA-deficiency (0.15-0.16 g/L, ref 0.70-4.00 g/L) with a strong increase in IgM (7.29-8.11 g/L, ref 0.40-2.30 g/L) and was further diagnosed with acute cutaneous, discoid and chilblain lupus erythematosus with borderline positivity of anti-nuclear antibodies at 3 years post-HSCT. Donor peripheral blood chimerism decreased gradually from 99% at 2 months to 44% in granulocytes and 30% in leucocytes (37% in CD19^+^ B, 53% in CD4^+^ and 56% in CD8^+^ T cells) at 3-years post-transplant, remaining stable since. There were no SCD complaints since transplant at a stable HbS of 9%. No anti-cytokine antibodies against IL-6, IL-17a, IL-22, IFN-α and IFN-γ were detected. Only HLA class-I antibodies were found pre- and post-HSCT and the donor’s IgG, IgA and IgM titers were normal, so there was no clear explanation for the persistent hypogammaglobulinemia and clinical complaints from the patient’s diagnostic workup.

## Methods

### Sample selection

This study incorporates longitudinal patient samples from peripheral blood and lymph node (LN), with approval from the Institutional Review Board of the LUMC-WAKZ (B17.001). All samples were stored in the LUMC biobank after informed consent. Peripheral blood mononuclear cells (PBMCs) from three healthy donors were sourced from the LUMC healthy voluntary donor service (LuVDS23.051) for use in this study.

### B-cell subsets

B-cell subsets analysis was performed on PBMCs as previously described (7). In brief, absolute counts of the following populations were identified: transitional B (CD19^+^CD38^hi^CD24^hi^); naïve mature B (CD19^+^CD38^dim^CD27^-^IgD^+^); marginal zone-like/natural effector B(MZ/NE; CD19^+^CD38^dim^CD27^+^IgD^+^); and memory B cells (MBC, CD19^+^CD38^dim^CD27^+^IgD^-^).

### *In vitro* B-cell stimulation

Naïve B cells (CD20^+^CD27^-^IgG^-^IgA^-^) were sorted from cryopreserved PBMCs of patient samples for *in vitro* B-cell stimulation as described previously (7). On day 0 of the culture, 50.000 plated naïve cells were stimulated with human recombinant MEGACD40L Protein (Fluka), IL-21 and IL-10 (PeproTech) and aIgM (Jackson ImmunoResearch) and restimulated on day 3 with all except aIgM.

On day 7, previously established methods were used to determine class-switching by flow cytometry and expression of activation-induced (cytidine) deaminase (AID) by real-time quantitative RT-PCR (RT-qPCR) (8). In parallel, the harvested supernatants were used to determine IgG and IgA levels by sandwich ELISA. Data were visualized in GraphPad Prism version 8 (GraphPad Software).

### Flow cytometry and cell-sorting of subsets from peripheral blood and lymph node

Transitional B, naïve mature B, MZ/NE, MBC, and follicular helper T cells were analyzed by flow cytometry and sorted from frozen PBMCs and lymph node mononuclear cells (LNMC) using specific fluorochrome-conjugated antibodies. For PBMCs, the antibodies included CD3 (UCHT1, BV421, BD Biosciences(BD)), CD38 (HIT2, BV605, Biolegend (BL)), CD24 (ML5, BV650, BD), CD19 (SJ25C1, BV786, BD), IgM (G20-127, BB515, BD), IgD (IA6-2, PerCP-Cy5.5, BL), CD45RA (MEM-56, PE-TxR, Invitrogen), CXCR5 (REA103, PE-Vio770, Miltenyi Biotec), CD27 (L128, APC, BD), and CD4 (RPA-T4, APC-H7, BD) and Brilliant Stain+ (BS+, BD) buffer diluted in FACS-buffer (PBS/0.5% BSA/2mM EDTA). For LNMCs, the antibodies used were CXCR4 (12G5, BV421, BD), IgM (MHM-88, BV510, BL), CD38 (HIT2, BV605, BL), CCR7 (G043H7, BV711, BL), CD19 (SJ25C1, BV786, BD), IgD (IA6-2, FITC, BL), PD1 (EH12.1, RB780, BD), CD27 (L128, PE, BD), CD45RA (MEM-56, PE-TxR, Invitrogen), CXCR5 (REA103, PE-Vio770, Miltenyi Biotec), CD21 (B-ly4, APC, BD), CD4 (SK3, AF700, BL), CD8 (RPA-T8, BUV395, BD) and CD3 (SK7, APC-H7, BD) and BS+ buffer diluted in FACS-buffer. Flow cytometry and sorting were performed on the Cytek^®^ Aurora (Cytek) and BD FACSAria™ III Cell Sorter (BD) respectively. Sorted populations were submitted to the laboratory for Specialized Hematology at the LUMC for chimerism analysis.

### B-cell receptor repertoire sequencing

B-cell receptor (BCR) repertoire sequencing was conducted following established procedures (9). In brief, RNA was isolated from total PBMCs and specific FACS-sorted LN fractions: 171507 naïve B cells, 1095399 centroblasts, 219649 centrocytes, 32786 MBCs, and 8835 plasma cells. 310x310 base pair sequencing was performed on the MiSeq™ System with the Nextera XT Library prep kit (Illumina). Raw reads were germline-allocated using IMGT and filtered for a Phred score of 25. Visualizations were created with Antigen Receptor Galaxy and Graphpad Prism and represent reads without introduced Ns and removal of duplicates based on ‘Top V, Top D, and Top J gene, identical complementarity determining region (CDR) 3-nucleotides’. *IGHM* reads of PBMCs were subdivided into naïve and MBCs based on having 0-to-2 or more than 2% somatic hypermutations (SHM).

## Results

### Persisting hypogammaglobulinemia *in vivo* cannot be explained by an intrinsic B-cell defect

To obtain insight into the mechanism of hypogammaglobulinemia in this patient detailed immune phenotyping and functional studies were performed. Detailed flow cytometric immunophenotyping revealed a significant increase of transitional and naïve mature B cells, with reduced numbers of marginal zone/natural effector (MZ/NE) cells and nearly absent memory B-cells (MBCs; **Table 1**). Because of the low MBC-count, further distinction between IgM^+^, IgG^+^ and IgA^+^ MBCs could not be made. Next, we sorted transitional B, MZ/NE, naïve mature B, and follicular helper T (Tfh) cells for chimerism analysis. In line with earlier observations, we found a chimerism-discrepancy, with B cells being 81-84% of patient and Tfh cells only 61% (**Table 1**).

**Table 1:** B- and T-cell counts and frequencies in peripheral blood and lymph node.

Peripheral blood	6 years pHSCT cells/µl	Normal values cells/µl(9)	Chimerism % patient
**B-cell subsets**			
** Transitional B** (CD38^hi^CD24^hi^)	178	3-50	84
** Naïve mature B** (CD38^dim^CD27^-^IgD^+^IgM^+^)	611	57-447	82
** MZ/NE** (CD38^dim^CD27^+^IgD^+^)	21	9-88	81
** Memory B** (CD38^dim^CD27^+^IgD^-^)	1	13-122	nd
**T-cell subsets**			
** Helper T** (CD3^+^CD4^+^)	1136	300-1400	nd
** Tfh** (CD4^+^CD45RA^-^CCR7^+-^CXCR5^hi^)	122		61
** Cytotoxic T** (CD3^+^CD8^+^)	1227	260-990	nd
Lymph node	6 years pHSCT %	Normal values average (range) %(9)	Chimerism % patient
**Naïve mature of total B** (CD19^+^CD21^+^CD38^dim^CD27^-^IgD^+^)	87.8	42.5 (29.2 - 57.5)	81
**GC of total B** (CD19^+^CD21^+^CD38^+^IgD^-^)	9.9	20.6 (11.2 - 32.0)	
Centroblast of GCB (CXCR4^hi^)	76.1	63.8 (54.4 – 76.2)	100
Centrocyte of GCB (CXCR4^lo^)	22.6	31.3 (20.3 – 38.5)	100
IgG^+^ of GCB	0.8	37.5 (16.1 – 57.9)	
IgA^+^ of GCB	0.0	26.3 (12.4 – 51.4)	
**MBC of total B** (CD19^+^CD21^+^CD38^dim^CD27^+^IgD^-^)	0.7	14.8 (6.30 – 24.0)	68
IgG^+^ of MBC	3.0	40.4 (18.9 – 57.0)	
IgA^+^ of MBC	0.1	25.3 (16.5 – 38.5)	
IgM^+^ of MBC	65.5	4.9 (1.75 – 10.7)	
**Plasma cell of total B** (CD19^+^CD21^+^CD38^hi^)	0.6	1.0 (0.40 – 1.90)	nd
**Helper T cells** (CD3^+^CD4^+^)	59.8		nd
** Tfh of total CD4^+^** (CD45RA^-^CCR7^+-^PD1^hi^CXCR5^hi^)	19.2		26
**Cytotoxic T cells** (CD3^+^CD8^+^)	31.5		nd

MZ/NE, marginal zone/natural effector cell; Tfh, T follicular helper cell; GC, germinal center; MBC, memory-B cell; nd, not determined.

To determine whether the hypogammaglobulinemia was caused by an intrinsic B-cell defect, we sorted naïve B cells and performed an *in vitro* B-cell stimulation experiment using class-switch recombination (CSR) as functional readout (**Figures 1B, C**). Over 7 days of stimulation, B cells proliferated, differentiated into class-switched IgG^+^ and IgA^+^ MBCs and secreted IgG and IgA in the supernatant (**Figures 1B–D**). Of note, we found a large proportion of IgM^+^ MBCs, although we could not reliably determine the production of IgM by ELISA due to prior stimulation with aIgM (**Figures 1C, D**). Normal CSR was further supported by the expression of *AID* by qPCR, although the difference in expression compared to HDs suggests a timing-bias compared to the strong increase in MBCs (**Figures 1B, C, E**). Collectively, these findings ruled out an intrinsic B-cell defect in CSR as grounds for the patients’ hypogammaglobulinemia.

### Follicular hyperplasia of secondary lymphoid organs with an absolute increase in IgM^+^ plasma cells and complete IgA deficiency

Given that naïve B cells demonstrated differentiation-capacity into class-switched MBCs *in vitro*, our focus shifted to the secondary lymphoid organs (SLOs) and germinal centers (GCs). Due to the patient’s variable yet persistent lymphadenopathy, a lymph node (LN) was excised and subjected to immunohistochemistry to rule out a infectious cause or malignancy.

**Figure 2:**
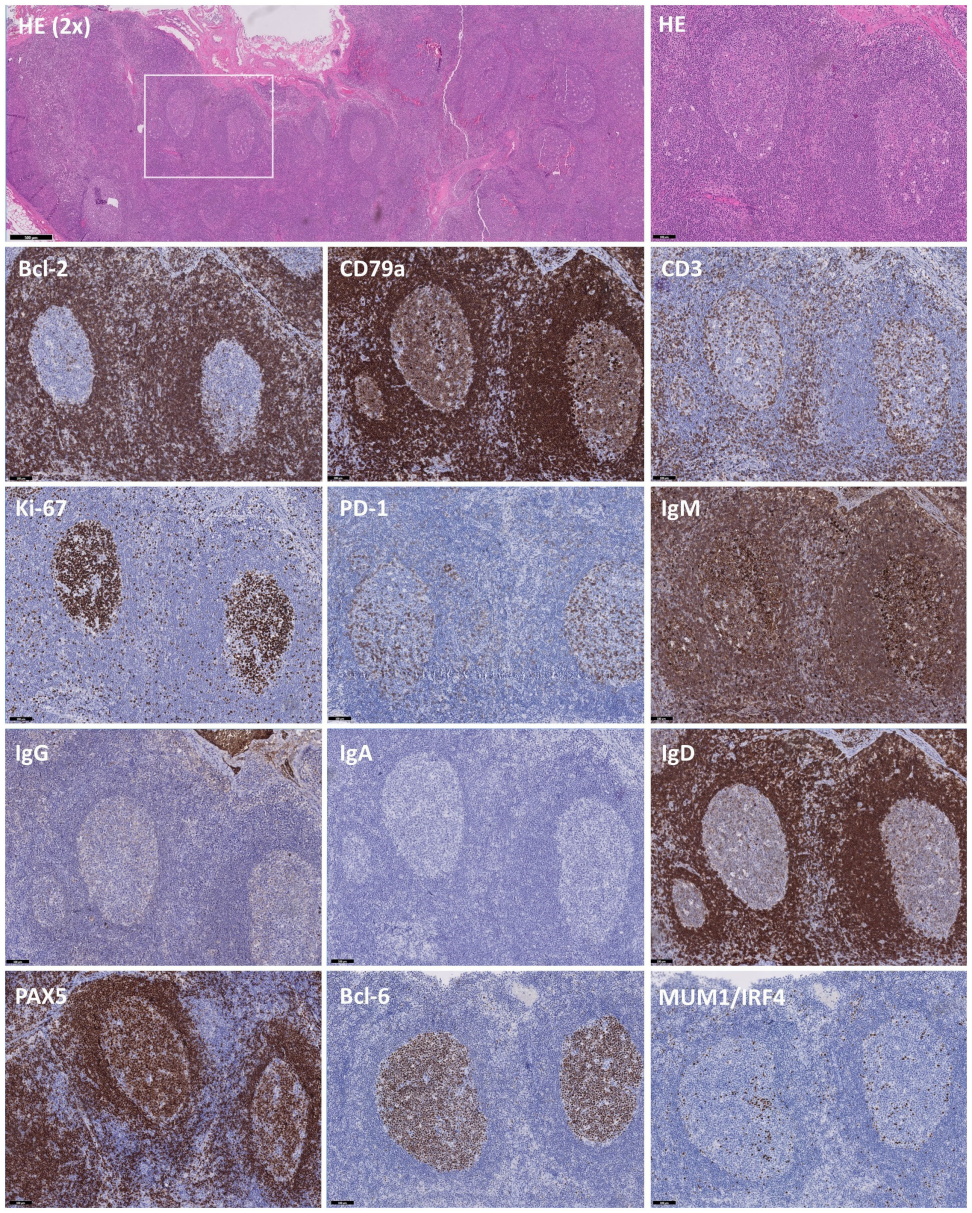
Immunohistochemistry of B and T cell-specific markers on slides of an excised lymph node. Immunohistochemistry on excised lymph node tissue with hematoxylin and eosin (HE), Bcl-2, CD79a, CD3, Ki-67, PD-1, IgM, IgG, IgA, IgD, PAX5, Bcl-6 and MUM1/IRF4, showing distinct staining patterns. HE (2X) staining showing follicles varying from normal to hyperplastic. The white box indicates the chosen area for further analysis. Predominant staining with IgM and scarcity of IgG and IgA-deficiency indicates a class-switch defect, further supported by sparse expression of MUM1/IRF4 in areas with low PAX5 and Bcl-6 staining. Images are at 10X magnification unless otherwise specified and validated against a healthy control.

Hematoxylin and eosin (HE) staining revealed increased and variably enlarged nodular follicles, with tangible body macrophages forming a characteristic starry-sky pattern (**Figure 2**). Bcl-2 and CD79a highlight the mantle zone, with a negative and moderately-positive GC staining, respectively. CD3 indicated numerous T cells in the interfollicular space, with few Tfh cells residing in the GC. Ki-67 expression showed a highly proliferative fraction within the GC, and PD-1 on Tfh cells demarcated the dark and light zones (DZ, LZ respectively). IgM expression was increased significantly, while IgG^+^ cells were reduced, and IgA staining was absent, which was consistent with the serum Ig levels of hyper-IgM and low IgG and IgA. IgD highlighted the mantle zone and was largely absent inside the GC, denoting naïve-to-MBC differentiation. A strong cytoplasmic signature of CD79a, downregulation of PAX5 and Bcl-6 and IRF4-expression evidenced the initiation of plasma-cell differentiation, albeit rare. In parallel, immunohistochemistry on an excised tonsil also showed follicular hyperplasia and increased IgM^+^ cells with low IgG and absent IgA staining (not shown). Importantly, EBER staining was negative in LN, ruling out an EBV-associated malignancy together with absence of M proteins (not shown).

In parallel to immunohistochemistry, flow cytometry on LN confirmed increased naïve B cells, while GC B cells (GCBCs) were decreased with normal centroblast and reduced centrocyte frequencies (**Table 1**). We could also confirm the reduction in IgG^+^ B cells and absence of IgA-switched B cells. Notably, IgM^+^ MBCs were strongly increased, indicating a block in class-switching but not in the overall development of MBCs. Chimerism analysis of these FACS-sorted populations revealed that GCBCs were 100% patient-origin, while naïve B cells were 81% patient-derived. Only 26-percent patient Tfh cells resided in the GC, yet 68% of MBCs were patient-derived, confirming autologous MBC development regardless of low autologous Tfh-support and showing that donor-derived B cells could undergo interfollicular differentiation (**Table 1**).

### Antigen-selected GC-output is skewed towards lower somatic hypermutations

**Figure 3:**
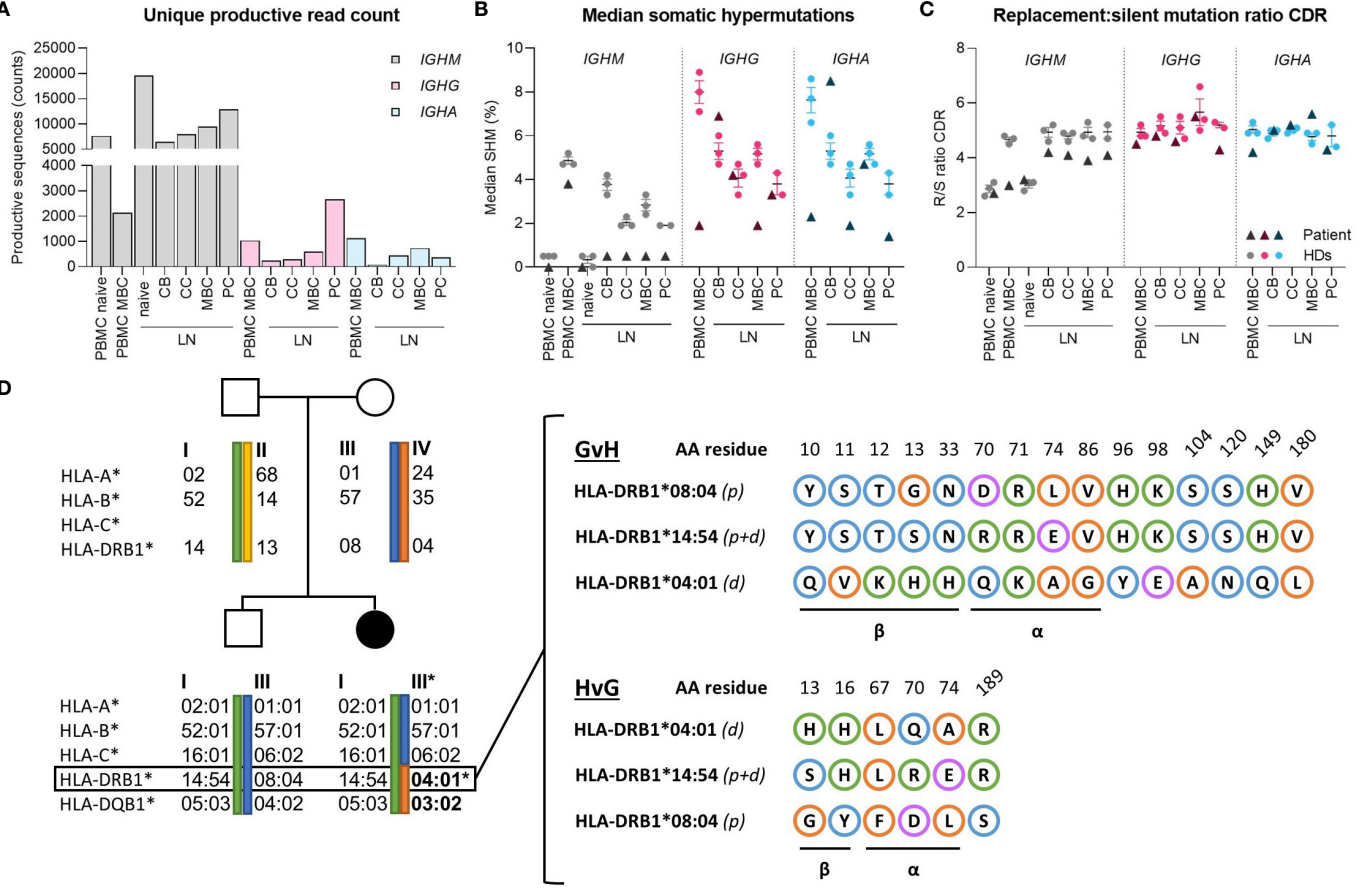
Molecular analysis of B-cell receptor repertoire and HLA-matching. **(A)** Absolute counts of productive reads from B-cell receptor repertoire sequencing after filtering for a Phred score of 25 and removing duplicates based on TopV, TopD, TopJ, CDR3 (nucleotides), respectively. For *IGHM* reads, the PBMC dataset was further subdivided into 0-2% and >2% SHM to represent naive and memory (MBC) subsets. **(B)** Median percentage SHM between *IGHM, IGHG* and *IGHA* of peripheral blood and lymph node. **(C)** Replacement-to-silent (R/S) mutation ratio in CDR of *IGHM, IGHG* and *IGHA* in peripheral blood and lymph node. **(D)** Family pedigree of patient showing the exact maternal translocation of HLA-II alleles and in-depth characterization of amino acid mismatches between patient and donor’s HLA-DRB1 alleles in the graft-versus-host and host-versus-graft direction. Blue, orange, green and purple circles indicate polar, non-polar, positively- and negatively-charged amino acids, respectively. CB, centroblast; CC, centrocyte; MBC, memory-B cell; PC, plasma cell; LN, lymph node; CDR, complementarity determining region.

To explore potential abnormalities in BCR-specification (somatic hyper mutations (SHM) and antigen selection), *IGHM*, *IGHG* and *IGHA* transcripts were amplified from PBMCs and sorted B-cell subsets from the LN. Only unique sequences were retained to preclude bias from clones or preferential BCR amplification, resulting in 66275, 4835 and 2746 sequences for *IGHM, IGHG* and *IGHA* respectively (**Figure 3A**). In peripheral blood, median SHM levels were normal in *IGHM* transcripts (**Figure 3B**), but strongly reduced in *IGHG* and *IGHA* transcripts compared to the van Schouwenburg et al. healthy donor (HD) data (9). In the LN, median SHM levels in *IGHM* were reduced in all GCBC-subsets, whereas the SHM levels in *IGHG* were only reduced in MBC and in centrocyte and plasma cell of *IGHA* (**Figure 3B**). Notably, *IGHG* and especially *IGHA* sequences were limited in number (**Figure 3A**). Despite undergoing SHM in the DZ, only *IGHG* and *IGHA* reads with limited SHM appeared to reach the periphery.

Evaluation of the replacement-to-silent (R/S)-mutation ratio in complementarity-determining regions (CDRs) revealed no differences in antigen selection between LN and PB compared to healthy donors (**Figure 3C**). Similarly, there was no difference in R/S ratio of the framework regions in unique productive sequences compared to HDs (not shown). Consequently, it can be inferred that affinity maturation through SHM and CSR can be induced at normal levels in the GC, despite a marked reduction in IgG^+^/IgA^+^ cells and reduced SHM of antigen-selected B cells in peripheral blood.

### Mixed chimerism with HLA class-II disparity

Although the B-cell intrinsic molecular processes of SHM and CSR were intact, the GC reaction appears to be insufficiently induced and/or impeded. As T-B interaction is pivotal in determining B-cell fate – selecting high-affinity GCBCs and inducing terminal B-cell differentiation (10, 11)– we explored the potential influence of the patient’s HLA class-II (HLA-II) disparity and chimerism status on the disrupted GC reaction.

Using the molecular matching tool HLA-EMMA (12), we assessed the MHC class-II (MHCII) mismatches based on the haplotype-amino acid (AA) sequence (**Figure 3D**). A total of 15 AA mismatches were identified in the graft-versus-host (GvH) and 6 in the host-versus-graft (HvG) direction in HLA-DRB1, of which 9 and 5 were localized within the α-helices and β-sheet (forming the antigen-binding cleft and base of the MHC molecule, resp.), respectively, determining the direct and indirect (allo)recognition by T cells and peptide repertoire presented in HLA (13). Similarly, the HLA-DQB1 mismatch harbors several bidirectional AA mismatches in the α-helices and β-sheet (not shown). These mismatches likely impede cognate T-B interaction in the GC between predominantly donor-Tfh and patient-B cells.

## Discussion

We describe a patient with persistent hypogammaglobulinemia, SLE-like skin lesions and lymphadenopathy after HSCT for SCD with mixed chimerism. We found no evidence of an intrinsic B-cell defect, because they expressed *AID*, could undergo SHM and were able to differentiate into IgG- and IgA-producing plasma cells *in vitro*. In the GC we found few, class-switched IgG+ plasma cells, indicating that terminal B-cell differentiation was not totally impaired but happened infrequently. Since all GCBCs were of patient origin, while only 26% of Tfh cells originated from patient, we hypothesize that the patient’s class-switch defect is related to HLA-II disparity limiting efficient T-B crosstalk between GCBCs and Tfh cells.

The patient had a translocation in the maternal HLA haplotype, receiving the HLA-II genes from the other maternal allele starting at HLA-DRB1. HLA class-I genes of patient and donor were fully matched, and at least half of the typed HLA-II alleles are matched (HLA-DRB1*14:54 and HLA-DQB1*05:03 of those tested). There is currently no evidence that HLA class-II disparity increases the risk for post-transplant hypogammaglobulinemia based on cohort studies on immunological recovery from mismatched related and unrelated grafts. In fact, mismatched related and unrelated donors were described to have similar B-cell recovery to matched related donors (14–16). Whether or not B-cell dysregulation occurs more frequently in patients with HLA class-II disparate HSCT and differential chimerism of T- and B-cell subsets remains to be determined.

Both patient and donor-derived naïve B cells were present in peripheral blood, indicating that the bone marrow microenvironment could support B-cell development of both patient and donor-derived B cells. Because we had no clinical indication to perform a bone marrow biopsy, we could not study the chimerism of B-cell precursors further. Analysis of bone marrow B-cell development can be especially informative in hypogammaglobulinemia cases coinciding with B-cell lymphopenia. Despite an ample supply of both patient and donor-derived naïve B cells in peripheral blood and LN, MBC-output was severely diminished. Naïve B cells were not intrinsically impaired in CSR *in vitro*, leading us to believe that terminal B-cell differentiation was impeded in the secondary lymphoid organs. In the LN, antigen-activated B cells migrate to the T-B border, where they activate CD4+ T cells. TCR signaling and ICOS-ICOSL crosslinking upregulates Bcl-6, CXCR5 and later IL-21, causing now pre-Tfh cells to mature and migrate into the GC (17). We could not deduce why Tfh cells were mainly donor-derived in the LN, but this must be due to an altered selection process.

Next to Tfh maturation, cognate TCR:pMHCII interaction also bifurcates B-cell differentiation into the extra-follicular (EF)- or GC-fate depending on the degree of T-cell help. A strong T-cell help signal is known to instigate GC formation and terminal differentiation of antigen-selected memory B cells, which depends on TCR:pMHCII crosslinking of multiple complexes (10, 18). The formation of GCs was not constrained by the noticeably skewed T:B chimerism, yet exclusively patient-derived B cells could initiate a GC reaction, even though 19% naïve donor B cells resided in the lymph node. The GCBC-fate, determined by intermediate T-cell help via CD40 and Bcl-6 was initiated by patient-derived B cells, but IRF4 expression was not sufficient to induce class-switching pre-GC maturation. Class-switching is dependent on strong T-cell help which facilitates *AID*-expression via IRF4 (19, 20), which we could normally simulate during B-cell differentiation *in vitro*. GC formation and architecture appeared normal in LN, and patient-derived GCBCs underwent normal proliferation and SHM compared to HDs in the DZ. In addition, antigen selection of the BCR repertoire was normal and apoptosis was not increased in the GC, indicating that cognate antigen on follicular dendritic cells (FDCs) provided GCBCs with the initial LZ-survival signal. Next, light-to-dark zone recirculation or differentiation into MBCs or plasma cells is again governed by T-cell help-levels.

Evidenced by sparse IRF4-expression, terminal plasma-cell differentiation was not blocked entirely but happened infrequently, indicating that patient GCBCs rarely received sufficient help from predominantly donor-derived Tfh cells (18, 21). While IgG^+^ but not IgA^+^ B cells were detectably by flow cytometry and immunohistochemistry, we found both *IGHG* and *IGHA* sequences by BCR repertoire sequencing. Based on the varying follicular hyperplasia of the lymph node tissue, it appears the remaining GCBCs become trapped in the GC, further increasing in size by the continual recruitment of additional activated B cells. While donor B cells did not initiate GCs they could form EF MBCs, a differentiation pathway that is not yet fully understood but likely results from weak T-cell help (18). Because MBCs in LN and peripheral blood had low SHMs and were mostly *IGHM*, we can conclude that patient and donor-derived B cells predominantly received weak T-cell help, via EF and/or GC responses.

HLA-compatibility plays a crucial role during the GC reaction, where high-affinity peptide-MHC-TCR interactions are necessary to induce terminal B-cell differentiation. HLA-II mismatches between centrocytes and FDCs may impede optimal antigen selection, although we would in principle expect 50% successful T:B interactions since at least the paternal HLA-II allotypes were matched between patient and donor. Evidently, haploinsufficiency alone might not be the sole explanation for the impediment in B-cell differentiation observed in this unique patient.

Based our investigations, we conclude that the patient’s hypogammaglobulinemia persisting for up to 7 years post-HSCT appears to result from inefficient T-B crosstalk inside the lymph node due to HLA-II disparity and skewed chimerism between T and B cells, although the exact mechanism remains elusive. We have shown that B-cell phenotyping of peripheral blood alone does not give the full picture of post-HSCT hypogammaglobulinemia; in depth cellular and molecular analysis of the GC reaction in secondary lymphoid organs can provide additional insights when studying these cases.

## Data availability statement

The IGH repertoire sequencing data have been deposited in the Gene Expression Omnibus (GEO) database and can be accessed through accession number GSE263200.

## Ethics statement

The studies involving humans were approved by the Institutional Review Board of the LUMC-WAKZ (B17.001). The studies were conducted in accordance with the local legislation and institutional requirements. Written informed consent for participation in this study was provided by the participants’ legal guardians/next of kin. Written informed consent was obtained from the individual(s) for the publication of any potentially identifiable images or data included in this article.

## Author contributions

MG: Conceptualization, Data curation, Formal analysis, Investigation, Methodology, Visualization, Writing – original draft, Writing – review & editing. IP-K: Investigation, Methodology, Writing – review & editing. MO-T: Investigation, Methodology, Writing – review & editing. DB: Writing – review & editing, Data curation. FS: Writing – review & editing, Data curation. AB: Writing – review & editing, Investigation, Methodology. HJ: Writing – review & editing, Data curation. PJ: Investigation, Methodology, Writing – review & editing. AL: Conceptualization, Supervision, Writing – review & editing. MB: Conceptualization, Methodology, Supervision, Writing – review & editing.
